# Prevalence of Irritable Bowel Syndrome and its Association with Anxiety among Medical Students at King Saud bin Abdulaziz University for Health Sciences in Riyadh

**DOI:** 10.12669/pjms.331.12572

**Published:** 2017

**Authors:** Meshal Khaled Alaqeel, Nasser Abdullah Alowaimer, Anas Fahad Alonezan, Nawaf Yousef Almegbel, Fahad Yousef Alaujan

**Affiliations:** 1 Dr. Meshal Khaled Alaqeel, Consultant Psychiatry and Assistant Professor, Department of Psychiatry, King Saud bin Abdul-Aziz University for Health Specialties, Riyadh, Saudi Arabia; 2 Dr. Nasser Abdullah Alowaimer, MBBS. Medical Interns, College of Medicine, King Saud bin Abdul-Aziz University for Health Specialties, Riyadh, Saudi Arabia; 3 Dr. Anas Fahad Alonezan, MBBS. King Saud bin Abdul-Aziz University for Health Specialties, Riyadh, Saudi Arabia; 4 Dr. Nawaf Yousef Almegbel, MBBS. King Saud bin Abdul-Aziz University for Health Specialties, Riyadh, Saudi Arabia; 5 Dr. Fahad Yousef Alaujan, MBBS. Medical Interns, College of Medicine, King Saud bin Abdul-Aziz University for Health Specialties, Riyadh, Saudi Arabia

**Keywords:** Anxiety, Medical students, Irritable Bowel Syndrome

## Abstract

**Objective:**

To quantify the prevalence of Irritable Bowel Syndrome (IBS) among medical students of King Saud bin Abdulaziz University for Health Sciences (KSAU-HS) and to observe the association between anxiety and IBS.

**Methods:**

This cross-sectional observational study conducted during academic year 2015-2016 has used two self-administered, pre-validated questionnaires: Depression Anxiety Stress Scales-21 (DASS-21) and Rome III criteria. The sample size was 270, and proportional allocation was used to determine distribution of this sample across study population based on percentages of students in each academic year. Convenience sampling was used to select participants.

**Results:**

The overall prevalence of IBS was 21% (n=57), with a higher prevalence among females (26%, n=23) than males (19%, n=34). IBS was most and least prevalent among first-year students (14%, n=5) and fifth-year students (29%, n=21) respectively. Anxiety levels were normal, mild, moderate, and severe or extremely severe in 39% (n=105), 7% (n=19), 26% (n=70), and 27%. A significant association was found between gender & IBS and anxiety levels & IBS.

**Conclusion:**

The prevalence of IBS in this study was 21% and higher among females than males but were highest among fifth-year students for both genders. More than 50% of students had moderate or high levels of anxiety for both genders. The prevalence of IBS was highest among students of 5^th^ fifth year. The study provides evidence that, as medical students of higher year of their under graduation were having higher level of anxiety which leads to IBS.

## INTRODUCTION

Irritable bowel syndrome (IBS) is a common medical disorder affecting the digestive tract in the gastrointestinal (GI) system. In the United States, the prevalence of IBS is 5-20%.^1^ IBS is more common among younger adults who are less than 50 years of age.^1^ The pathogenesis of this disease is not fully understood. However, there are many etiological explanations for this condition, with the main explanations involving inflammation and immunological factors.^2^

The relationship between psychiatric disorders and GI disorders such as IBS is well established.^3^,^4^ IBS patients typically suffer from anxiety and depression, which can aggravate their IBS symptoms. This phenomenon is attributable to the fact that the large intestine is partially controlled by the autonomic nervous system, which responds to stress.^5^ Comparisons between healthy individuals and IBS patients have demonstrated that stress increases the motility and sensation of the large intestine in individuals affected by IBS.^6^ Medical students face a substantial amount of stress as they attempt to fulfill their duties. Given the number of years required to graduate, frequent exams, long study hours, and need to work with difficult patients associated with medical school, medical students also have greater levels of stress which leads to higher prevalence of IBS than students in other disciplines.^7^

A Korean study found that the prevalence of IBS was 29.2% among 319 medical students.^8^ In Karachi, Pakistan, a case-control study involving diagnosing IBS was conducted in three medical schools; in this study, the prevalence of IBS was found to be up to 28%, with a significant difference between students in pre-clinical years and students in clinical years.^9^

In Saudi Arabia, studies conducted on medical students and interns in Jeddah reported a prevalence of IBS of 31.8%.^10^ Notably, there is a lack of studies on this specific topic in the Gulf area in general and in Saudi Arabia in particular. Hence this study was conducted among medical students at King Saud bin Abdulaziz University for Health Sciences (KSAU-HS) to quantify the prevalence of IBS and its association with anxiety among these students.

## METHODS

This cross-sectional study was conducted in the medical college of KSAU-HS during the 2015-2016 academic years. The study included first- to fifth-year medical students of both genders. Based on calculations performed using the Raosoft website, 270 out of the total population of 901 medical students were included (with a 95% CI, a 5% margin of error, and a response distribution of 50%).^11^ The sample was divided into 10 groups based on academic year and proportional allocation by gender. Participants were approached using convenience sampling. All participants provided informed consent. This study was approved by the Institutional Review Board of King Abdullah International Medical Research Center. Two self-administered and validated questionnaires were used. The first questionnaire is the Rome III criteria, a system developed to classify functional GI disorders (FGIDs), which are disorders of the digestive system for which clinical symptoms cannot be explained by the presence of structural or tissue abnormalities.^12^ The second questionnaire is the Depression Anxiety Stress Scales-21 (DASS-21), a set of three self-report scales designed to measure the negative emotional states of depression, anxiety and stress. The internal consistency of this scale which was measured by using cronbach’s alpha from our data shows 0.974 (95% Confidence interval: 0.969 to 0.978).

Data were analyzed using Statistical Package for Social Sciences (SPSS), version 22.0 (IBM Inc., Chicago, USA). Descriptive statistics (frequencies and percentages) were used to describe the categorical study and outcome variables. Pearson’s Chi-square test was used to observe the association between categorical study variables (gender, year study and anxiety levels) and outcome variable (presence/absence of IBS). A p-value of <=0.05 was used to report the statistical significance of results.

## RESULTS

Based on the Rome III criteria, 57 students were identified as having IBS, with a corresponding prevalence of 21.1%. With respect to the prevalence of IBS for students of different genders, the study results indicated that 19% of males and 25.9% of females had IBS With respect to the prevalence of IBS across students of different academic years; the highest prevalence was among fifth-year students, whereas the lowest prevalence was among first-year students (Table-I).

**Table-I:** Prevalence of IBS in all study subjects, male & female subjects and in relation to their year of study.

	Prevalence of IBS No. (%)
Among all study subjects (n=270)	57(21.1)
***Gender***	
Male (n=182)	34(18.6)
Female (n=88)	23(25.9)
***Year of study***	
First (n=39)	5(14)
Second (n=41)	10(26)
Third (n=40)	7(17)
Fourth (n=69)	14(20)
Fifth (n=81)	21(29)

The DASS-21 questionnaire was administered to students to assess their anxiety levels. The DASS-21 results revealed that 39%, 7%, and 25.9% of students had normal, mild, and moderate anxiety, respectively. Moreover, 13.3% and 14.1% of students had severe and extremely severe levels of stress, respectively (Table-II).

**Table-II:** Prevalence of Anxiety levels among the study subjects (n=268).

Anxiety levels	Prevalence No. (%)
Normal	105(39.2)
Mild	19(7.1)
Moderate	70(26.1)
Severe	36(13.4)
Extremely Severe	38(14.2)

A statistical significant association was observed between (i) gender and IBS (Chi-square = 3.94; p=0.047), and (ii) between IBS and the presence of anxiety (Chi-square = 10.06; p=0.039), but no statistical significant association was observed between year of study and IBS (Chi-square = 3.34; p=0.502).

## DISCUSSION

Highly diverse findings regarding the prevalence of IBS have been obtained in different studies. The difference may be due to the use of different study populations across countries and different diagnostic criteria. In the West, a study conducted in Canada on medical students with overnight shifts revealed a prevalence of IBS of 20.5%, which was similar to the overall prevalence that we determined.^13^ In the Far East, a study in Malaysia and an investigation in China reported lower prevalence of 15.8% and 15.7%, respectively.^14^ In contrast, one study in Korea and another investigation that included medical and nursing students in Japan reported higher prevalence of 29.2% and 35.5%, respectively.^7^,^8^ In Pakistan, the prevalence of IBS was determined to be 28.3%, which was higher than the prevalence found in the current study.^9^ In the Middle East, a study conducted in Lebanon that included students from different majors reported a prevalence of 20.6%, with students from health specialties accounting for half of IBS cases.^9^ Locally, a study in Jeddah on both medical students and interns revealed a prevalence of IBS of 31.8%. These differences in prevalence could be due to cultural, ethnic, and dietary habits in various countries and may also be attributable to the sample sizes, age groups, and diagnostic criteria used by different investigations.^10^

With respect to academic year, the current study demonstrated that IBS was most prevalent among final-year students, followed by second-year students. These high rates could be related to the increasing work load of fifth-year students and the introduction of a block system for second-year students. One local study conducted in Jeddah produced the same result with respect to the prevalence of IBS being highest among students in their final year.^10^ In contrast, a systematic review from Iran indicated that the prevalence of IBS was highest among first- and second-year students.^15^ An investigation in Ontario, Canada, revealed no significant difference between pre-clinical and clerkship students with respect to the prevalence of IBS.^13^

This study measured the prevalence of IBS for students of different genders; the results demonstrated that IBS was more prevalent among females, a finding consistent with those of other studies performed worldwide.^7^,^8^,^16^-^19^

With respect to anxiety levels, the aforementioned study in Pakistan reported that psychological symptoms of anxiety were encountered in 55.8% of participants with IBS, the majority of whom were female (84.2%).^9^ The prior study in Japan reported that individuals with IBS had higher scores on the Hospital Anxiety and Depression Scale (HADS) than control subjects.^7^ The investigation in Jeddah reported borderline anxiety and morbid anxiety in 31.1% and 36.1% of the included students, respectively.^10^

The association between psychological disorders and functional bowel disorders is well established, and anxiety is no exception.^3^,^4^ The results of this study also provide evidence of a significant association between IBS and anxiety. Multiple studies conducted worldwide have also demonstrated that individuals diagnosed with both IBS and high levels of anxiety experience an increased incidence of IBS symptoms compared with their peers.^9^,^10^,^14^,^20^,^21^

## CONCLUSION

The overall prevalence of IBS in this study was 21% and prevalence of IBS were higher among females than among males but were highest among fifth-year students for both genders. More than 50% of students had moderate or high levels of anxiety. There was a significant association between IBS and anxiety, a finding similar to the results of other studies worldwide. Stress management activities and student counseling sessions are recommended to decrease anxiety levels and to enable students to cope with different stressors during their academic lives.

### Limitations

The study findings could be considered, by taking into account the response bias and recall bias as the data was collected by using questionnaires.
